# The IL-23/IL-17 Pathway in Inflammatory Skin Diseases: From Bench to Bedside

**DOI:** 10.3389/fimmu.2020.594735

**Published:** 2020-11-17

**Authors:** Taoming Liu, Sheng Li, Shuni Ying, Shunli Tang, Yuwei Ding, Yali Li, Jianjun Qiao, Hong Fang

**Affiliations:** Department of Dermatology, The First Affiliated Hospital, Zhejiang University School of Medicine, Hangzhou, China

**Keywords:** IL-17 family, IL-23, IL-23/IL-17 axis, psoriasis, targeted therapy

## Abstract

Interleukin-17 (IL-17) is an essential proinflammatory cytokine, which is mainly secreted by the CD4^+^ helper T cells (Th17 cells) and subsets of innate lymphoid cells. IL-17A is associated with the pathogenesis of inflammatory diseases, including psoriasis, atopic dermatitis, hidradenitis suppurativa, alopecia areata, pityriasis rubra pilaris, pemphigus, and systemic sclerosis. Interleukin-23 (IL-23) plays a pivotal role in stimulating the production of IL-17 by activating the Th17 cells. The IL-23/IL-17 axis is an important pathway for targeted therapy for inflammatory diseases. Emerging evidence from clinical trials has shown that monoclonal antibodies against IL-23, IL-17, and tumor necrosis factor are effective in the treatment of patients with psoriasis, atopic dermatitis, hidradenitis suppurativa, pityriasis rubra pilaris, pemphigus, and systemic sclerosis. Here, we summarize the latest knowledge about the biology, signaling, and pathophysiological functions of the IL-23/IL-17 axis in inflammatory skin diseases. The currently available biologics targeting the axis is also discussed.

## Introduction

Interleukin-17A (IL-17A) is cloned from a T cell hybridoma activated in rodents (1) and is related to several immune-mediated disorders, such as autoimmune (2), oncogenic (3), and infectious (4) diseases. The T helper 17 (Th17) cells constitute a unique subset of CD4^+^ T cells and are the major source of IL-17 (5). IL-17A triggers cellular reactions not only in the keratinocytes, but also in some other cells, including neutrophils, endothelial cells, fibroblasts, and osteoclasts (6–10). In keratinocytes, the binding of IL-17A to IL-17 receptor (IL-17R) A, IL-17C, or IL-17RD stimulates keratinocyte proliferation. Subsequently, the release of inflammatory mediators and chemokines leads to inflammatory reaction (11, 12).

The cytokines, interleukin-23 (IL-23) and IL-17, have been confirmed to markedly affect chronic inflammation (10, 13–16). In addition, the discovery of the IL-23/IL-17 pathway has contributed to a clearer understanding of the underlying mechanism of inflammatory diseases. At present, therapies for inflammatory diseases have advanced from general immunosuppression to biologics against the IL-23/IL-17 signaling pathway, such as IL-17, IL-12/23 and IL-23 inhibitors. In this review, we highlight the potential implications of dysregulation of the IL-23/IL-17 axis in chronic inflammatory skin diseases, including psoriasis, hidradenitis suppurativa (HS), atopic dermatitis (AD), alopecia areata (AA), pityriasis rubra pilaris (PRP), pemphigus, and systemic sclerosis (SSc).

## Search Strategy and Selection Criteria

In this review, we are not intended to comprehensively review all pathways identified through human and murine laboratory studies or all clinical trials and case series in various inflammatory skin diseases. Nevertheless, we intent to focus on those targets of IL-23/IL-17 pathway demonstrated to be effective or potentially effective for treating human inflammatory skin diseases. We searched the published literature from PubMed and ClinicalTrial.gov with the search terms including ‘IL-17,’ ‘psoriasis,’ ‘atopic dermatitis,’ ‘hidradenitis suppurativa,’ ‘alopecia areata,’ ‘pityriasis rubra pilaris,’ ‘pemphigus,’ ‘systemic sclerosis,’ ‘secukinumab,’ ‘ixekizumab,’ ‘brodalumab,’ ‘bimekizumab,’ ‘ustekinumab,’ ‘tildrakizumab,’ ‘guselkumab,’ and ‘risankizumab’. We mainly focused on publications written in English between September 1, 2010, and September 15, 2020. We chose the references depending on the basis of their originality and relevance to the topic.

## IL-17 Signaling in Inflammatory and Autoimmune Diseases

Th17 cells are known to play an important role in inflammatory and autoimmune diseases (17–19). In general, IL-23 is involved in the activation of Th17 cells to induce the production of IL-17A, IL-17F, tumor necrosis factor (TNF), and IL-6 (20). Binding to its receptors, IL-23 contributes to the phosphorylation of receptor-associated JAKs and specific Tyr residues, and this is followed by activating the transcription of IL-17 and other genes. The participation of IL-23 is crucial in the differentiation of IL-17-expressing phenotypes, *via* activating the transcription factor retinoid-related orphan receptor-γt (ROR-γt) and signal transducer and activator of transcription 3 (STAT3) (21–23).

**Figure 1:**
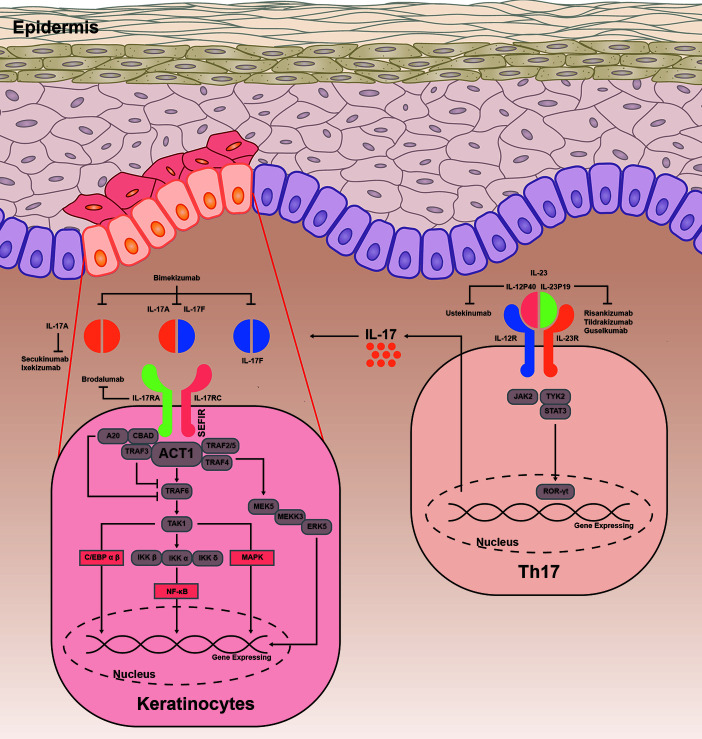
IL-23/IL-17 signaling transduction. IL-23 is important in differentiation of Th17 cells, by promoting the production of IL-17A, IL-17F, TNF, and IL-6. IL-23 is heterodimeric and composed of IL-12p40 and IL-23p19. Binding to its receptors, IL-23 involves in phosphorylation of JAKs and TYK, as well as phosphorylation and dimerization of STAT3. Subsequently, STAT3 homodimers regulates the expression of ROR-γt to promote the gene expression. The combination of IL-17A/A, IL-17A/F, or IL-17F/F cytokine with IL-17RA and IL-17RC is found to be a crucial complex of immune response. IL-17R acts on Act1 through interaction platform of the SEFIR domain. Upon ligand binding, Act1 activates NF-κB, C/EBP family, and MAPK pathway by inducing various TRAF proteins. Act1 is essential for mediating ubiquitination of TRAF6, then TRAF6 triggers a positive reaction in multiple different pathways. TRAF6 recruits and stimulates the TAK1 and IKK complex, leading to activation of NF-κB pathway. IL-17R-Act1 complex together with TRAF4, MEKK3, and MEK5 to promote activation of ERK5. In addition, ACT1-TRAF2-TRAF5 complex is capable to maintain the mRNA stability targeting the IL-17 gene. The inhibitors A20 and TRAF3 are linked with IL-17RA, dependent on the CBAD. C/EBP, CCAAT/enhancer-binding proteins; NF-κB, canonical nuclear factor-κB; MAPK, mitogen-activated protein kinase; TRAF, tumor necrosis factor receptor associated factor; TAK1, transforming growth factor-β activated kinase 1; IKK, inhibitor of kappa B kinase; ERK5, extracellular signal-regulated kinase 5; RORγt, retinoid-related orphan receptor-γt; STAT3, signal transducer and activator of transcription 3; JAK2, Janus activated kinase 2; TYK2, tyrosine kinase 2.

IL-17 induces expression of downstream genes by stimulating activation of pathways, including canonical nuclear factor-κB (NF-κB), CCAAT/enhancer-binding protein (C/EBP) family, and mitogen-activated protein kinase (MAPK) (**Figure 1**). The key complex, which is consisted of IL-17A/A, IL-17A/F, or IL-17F/F cytokine and IL-17RA or IL-17RC, is the start hallmark of IL-17 signaling transduction (24, 25). Moreover, IL-17RD is also found to be a functional receptor for IL-17A groups. Together with IL-17RC, IL-17RD acts on the downstream of proinflammatory gene expression of IL-17 signaling (12). IL-17R is characterized by a unique structure in its cytoplasmic tail, termed SEF/IL-17R (SEFIR) domain (26). IL-17 signaling recruits Act1 to IL-17R through interaction platform of SEFIR domain (27). Then Act1 (also known as an E3 ligase) promotes activation of distinct downstream signaling cascades by tumor-necrosis factor receptor–associated factor (TRAF) 6 (28). TRAF6 then recruits and stimulates the transforming growth factor β-activated kinase 1 and the inhibitor of kappa B kinase complex, resulting in activation of NF-κB, C/EBPβ, C/EBPδ, and MAPK pathway (29–31). IL-17R-Act1 complex binds with MEKK3 and MEK5, leading to keratinocyte proliferation (32). Act1 binds with TRAF2-TRAF5 to maintain the mRNA stability targeting IL-17 gene (33). In contrast, TRAF3 triggers a negative reaction in activation of NF-κB and MAPK pathway, resulting in suppressing the formation of IL-17R-Act1-TRAF6 (34). TRAF6, in combination with A20 (an anti-inflammatory protein) when presented, blocks the activation of NF-κB and MAPK to negatively regulate IL-17 signaling (35).

## Psoriasis

### Role of IL-17 Family Members in Psoriasis

In patients with psoriasis, the IL-17 concentrations increase not only in the skin lesions and peripheral blood, but also in the nonlesional and uninvolved skin (36–40). There is evidence indicating that the main sources of IL-17A in patients with psoriasis are the neutrophils (41), Th17 cells (42), mast cells (43, 44), CD8^+^ T cells (45), αβ T (46), γδ T cells (47), and innate lymphoid cells (48, 49) in the skin lesions.

**Figure 2:**
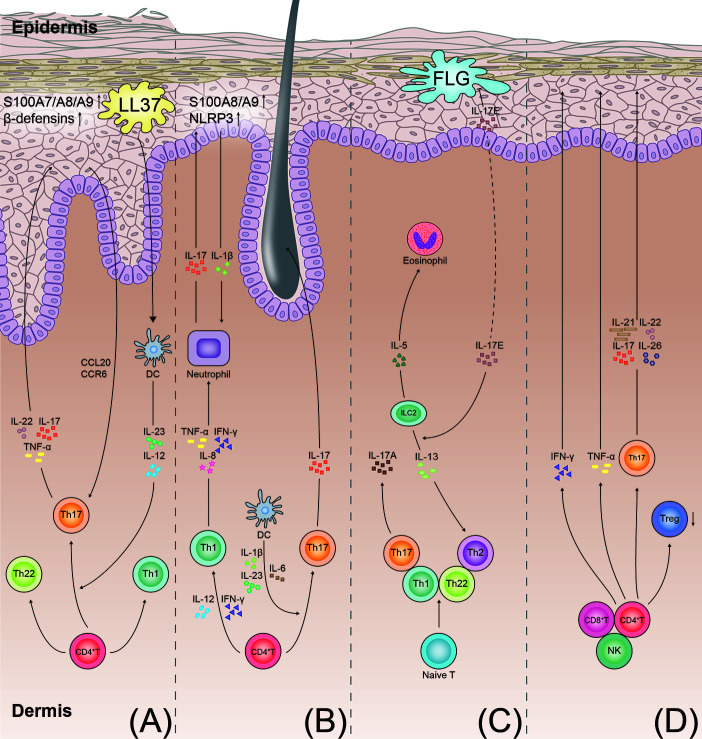
T-cell immune axis and associated cytokines in the pathogenesis of psoriasis, hidradenitis suppurativa, atopic dermatitis, and alopecia areata. **(A)** Psoriasis develops through the aberrant activation of the dendritic cells producing IL-12 and IL-23. The dendritic cells induce the differentiation of the Th 17 cells and Th1 cells. IL-23 promotes the Th17 cells to secrete IL-17, IL-22, and TNF-α. In keratinocytes, IL-17 also stimulates production of antimicrobial peptides (S100A7/A8/A9 proteins and beta defensins). These cytokines promote keratinocyte proliferation and neutrophil recruitment, resulting in the formation of psoriatic plaques. **(B)** In hidradenitis suppurativa, the T cells involved in the pathogenesis of hidradenitis suppurativa include the Th1 and Th17 cells. IL-23 induces the differentiation of the Th17 cells and overexpression of IL-17. IL-17 induces the expression of the proinflammatory proteins (S100A8/A9) and NLRP3 in the keratinocytes. More inflammatory cytokines are recruited to the follicular unit and perilesional skin. **(C)** In atopic dermatitis, with impairment of the skin barrier in patients with atopic dermatitis, the damaged keratinocytes produce inflammatory cytokines (IL-17E). The cytokines stimulate the ILC2s to secrete type 2 cytokines (IL-5 and IL-13). IL-17E also inhibits the synthesis of FLG. IL-4, IL-13, and IL-31 directly stimulate the sensory nerves to promote pruritus. **(D)** In alopecia areata, the elevated IFN-γ levels in the perifollicular area activate the differentiation of the CD4^+^ T cells into various types of T cells, as shown. Th17 cells act on the hair follicle by producing proinflammatory mediators (IL-17, IL-21, IL-22, and IL-26), ultimately leading to the disruption of hair growth. TNF, tumor necrosis factor; NLRP3, NACHT, LRR, and NACHT, LRR, and PYD domains-containing protein 3; FLG, filaggrin; ILC2s, type 2 innate lymphoid cells; DC, dendritic cell.

Psoriasis autoantigens, such as LL37 (50), NFKBIZ (51), ADAMTSL5 (52), and CARMA2 (53), play a crucial role in the production of IL-17A and are involved in the pathogenesis of psoriasis. In psoriasis, the combination of LL37 with the patient’s own DNA leads to the activation of the Toll-like receptor 9 (54). The self-DNA-LL37 complex acts on Toll-like receptor 7 in the plasmacytoid dendritic cells (DCs) and triggers the activation of the classical myeloid DCs (55) (**Figure 2**). Subsequently, the myeloid DCs produce IL-12 and IL-23. IL-23 induces the differentiation of the CD4^+^ T cells into the Th1 cells and Th17 cells by stimulating the transcription factor ROR-γt and STAT3 (56, 57). Thereafter, the activated Th17 cells secrete Th17 cytokines (IL-17A, IL-22, and TNF-α), leading to the development of a positive feedback loop. IL-17 plays a key role in stimulating the NF-κB and MAPK signaling pathways (53, 58), contributing to a high expression of proinflammatory factors (CC-chemokine ligand 20 and CC-chemokine receptor 6) (14, 38). These proinflammatory cytokines and chemokines recruit the inflammatory cells and stimulate keratinocyte proliferation (59).

IL-17A plays an essential role in inflammation, metabolism, and bone/joint damage (8, 10, 15, 60–62). The IL-17 levels and Th17 cell frequencies are high in the skin, synovial fluid, and synovium tissue of the patients with psoriatic arthritis (PsA) (18, 63–65). In the synovium of patients with PsA, the mast cells and CD8^+^ T cells are the main sources of IL-17A (66). The aberrant expression of IL-17A directly affects the osteoclast precursors, leading to bone destruction in PsA (67). In addition, IL-17A interacts with the mediators of the Wnt signaling pathway in the osteoblasts and osteocytes, thus, preventing bone formation (10). Psoriatic inflammation is not restricted to the skin or joints. Metabolic disorders, such as hyperglycemia (68) and cardiovascular risks (69), are also associated with psoriasis.

Apart from IL-17A, the other IL-17 family members (IL-17C, E, and F) may also be involved in the pathogenesis of psoriasis (70–72). In contrast to that in the nonlesional psoriatic skin, mRNA expression of IL-17A, IL-17C, IL-17E, and IL-17F is increased in the lesional psoriatic skin (39, 70, 73). The IL-17C and IL-17E levels are higher than the IL-17A levels in the skin lesions in the psoriatic animal models (7, 73). Through the STAT3 pathway, the binding of IL-17E to IL-17RB induces keratinocyte proliferation to amplify skin inflammation in the psoriatic animal models (70). However, the effects of IL17-C on the mechanisms of psoriasis are not clear. Both IL-17F and IL-17A share homomeric and heterodimeric proteins with 50% sequence identity. The molecular structure and function of IL-17F are highly similar to those of IL-17A (74). Bimekizumab (an inhibitor of both IL-17A and IL-17F) is more effective than a blockade of IL-17A or IL-17F alone, especially for suppressing neutrophil chemotaxis and activating the synoviocytes or human dermal fibroblasts *in vitro* (71).

### Targeted Therapy in Psoriasis

The targeted biologics in the treatment of psoriasis are listed in **Supplementary Table**. Biologics targeting IL-23 or IL-17A have shown remarkable effects in the treatment of psoriasis. The anti-IL-17 agents approved by the FDA include secukinumab (anti-IL-17A), ixekizumab (anti-IL-17A), brodalumab (anti-IL-17RA), and bimekizumab (anti-IL-17A and -17F). Biologics against IL-23 include ustekinumab (anti-IL-12/23p40), tildrakizumab (anti-IL-23p19), guselkumab (anti-IL-23p19), and risankizumab (anti-IL-23p19).

### Targeting IL-17

#### Secukinumab

Secukinumab, a human immunoglobulin G1 monoclonal antibody against IL-17A, is an effective and safe biologic for psoriasis, involving skin, nails (75), and PsA (76). The data from the phase III randomized trials (ERASURE and FIXTURE) showed that secukinumab at doses of 300 or 150 mg is effective and safe for the treatment of moderate-to-severe psoriasis up to week 52 (**Supplementary Table**) (77). Secukinumab maintains significant powerful and long-lasting effects on the patients receiving the 300 mg secukinumab treatment every 4 weeks. The Psoriasis Area and Severity Index (PASI) 90/100 was 66.4%/41% at 156 weeks (78).

In the FUTURE 2 study, patients with PsA receiving secukinumab therapy achieved excellent and sustained improvement in the PASI90 and American College of Rheumatology 50 (ACR50) response at week 24 (**Supplementary Table**) (79). The results for patients who continued the study showed that, at week 104, the ACR50 response rates were 50.6 and 36% with the 300 and 150 mg doses of secukinumab, respectively (80). Secukinumab is important in suppressing synovitis and structural bone changes in patients with PsA at week 24, and low rates of radiographic progression are maintained at week 52 with secukinumab (81, 82).

Secukinumab has significant and long-term efficacy for the treatment of nail psoriasis. Therefore, the improvement in the Nail Psoriasis Severity Index (NAPSI) in the secukinumab 300 and secukinumab 150 groups was 73 and 63.6% at week 128, respectively (75). In summary, secukinumab shows excellent and sustained efficacy for the treatment of patients with moderate-to-severe plaque psoriasis and patients with psoriasis with or without arthritis and nail involvement.

#### Ixekizumab

Ixekizumab, a humanized immunoglobulin G4 monoclonal antibody, selectively blocks IL-17A. A multicenter trial (UNCOVER-3) reported that ixekizumab shows long-term efficacy for treating moderate-to-severe plaque psoriasis and that the treatment effects are strongly sustained for up to 156 weeks (**Supplementary Table**) (83, 84). Similarly, ixekizumab maintains promising clinical improvements in the scalp, nails, and palm (83).

According to the data from phase III studies (SPIRIT-P1 and SPIRIT-P2), ixekizumab is associated with improvements in disease prognosis and physical function in patients with active PsA, particularly, in those who are refractory to therapies or have an inadequate response to the anti-TNF therapies (**Supplementary Table**) (85, 86). To date, blockade of IL-17A is being advocated as first-line for treatment of PsA by the European League Against Rheumatism in 2019 (87). In general, the aforementioned studies suggest that ixekizumab is effective in controlling psoriasis and PsA, particularly in patients with lesions in hard-to-treat areas or in those who are refractory to treatments.

#### Brodalumab

Brodalumab, a fully human immunoglobulin G2 IL-17RA antagonist, leads to a rapid improvement in the molecular, histological, and clinical features of psoriasis at week 12 (88). The AMAAGINE-1 study showed that brodalumab shows sustained efficacy (120 weeks) in the treatment of moderate-to-severe plaque psoriasis (**Supplementary Table**) (89, 90). Brodalumab inhibits a broader range of targets, namely, IL-17AA, IL-17AF, IL-17FF, IL-17C, and IL-17E *via* IL-17RA, compared with secukinumab and ixekizumab. An open-label study involving 39 patients with moderate-to-severe psoriasis revealed that brodalumab treatment may be effective for the patients who did not respond to secukinumab, ixekizumab, or ustekinumab (91, 92).

Moreover, the data from an open-label study have indicated significant beneficial effect of brodalumab on psoriatic erythroderma (n = 18) and generalized pustular psoriasis (n = 12; **Supplementary Table**) (93). However, the sample size of this study was small; hence, multicenter trials with large sample sizes of patients with psoriatic erythroderma or generalized pustular psoriasis should be conducted.

#### Bimekizumab

Bimekizumab, a humanized monoclonal IgG1 antagonist neutralizing both IL-17A and IL-17F, is effective for PsA and moderate-to-severe plaque psoriasis (71, 94). Two phase II trials (BE ABLE 1 and BE ABLE 2) reported the safety and efficacy of bimekizumab for the treatment of moderate-to-severe plaque psoriasis (**Supplementary Table**) (94, 95). Patients with active PsA, who were administered bimekizumab, showed marked improvements in their condition at week 48 (**Supplementary Table**) (96).

### Adverse Events of Targeting IL-17 Therapy

The most commonly noted treatment-emergent adverse events (TEAEs) are infections, nasopharyngitis, headache, and diarrhea in patients treated with IL-17 inhibitors compared with those treated with a placebo (76, 97–99). A systematic review speculated that it was safe to use IL-17 antagonists (secukinumab, ixekizumab, and brodalumab) for patients with psoriasis with latent tuberculosis infection (100). However, eczematous eruptions were reported in some patients after treatment with biologics against IL-17A (secukinumab or ixekizumab) for 3–4 months (101). To date, the mechanism underlying the onset of eczematous adverse events after anti-IL-17A treatment is not clear. Both the Th1 and Th2 responses are involved in the pathogenesis of eczema. This may be due to the anti-IL-17 biologics mainly inhibit the Th17 cytokines and mediate an imbalance in the Th2/Th17 immune response, thus leading to eczematous eruptions (102–104). The deficiency of Th17 cells, IL-17RA, and IL-17F are essential for host defense against fungal pathogens in mucocutaneous and oral epithelial cells (105–109). The risk of chronic mucocutaneous candidiasis increases in patients received IL-17 blockades (secukinumab, ixekizumab, brodalumab, or bimekizumab) (94, 96, 110).

### Targeting IL-23

Ustekinumab, a humanized IgG1 monoclonal antibody against the p40 subunit of IL-12 and IL-23, is approved for treating adult and pediatric patients with moderate-to-severe plaque psoriasis (111). Two phase III trials (PHOENIX 1 and PHOENIX 2) reported rapid and sustained efficacy of ustekinumab when administered at doses of 45 mg or 90 mg every 12 weeks for patients with moderate-to-severe plaque psoriasis (**Supplementary Table**) (112, 113).

According to the phase IV trial (VIP-U), ustekinumab may reduce aortic vascular inflammation transiently (at week 12) and downregulate the expression of the inflammatory cytokines (TNF-α, IL-1β, IL-17A, IL-18, and IL-6) sustainably (at week 52) (114). In 25 patients with psoriasis, inflammation in the liver, spleen, and artery decreased after treatment with ustekinumab, as indicated by the radiography findings (115). A summary analysis indicated that ustekinumab has long-term (5 years) safety with respect to patients with moderate-to-severe psoriasis (116).

Risankizumab, a humanized IgG1 monoclonal antibody inhibits the p19 subunit of IL-23 (117). In the landmark UltIMMa-1 and UltIMMa-2 studies, 150 mg risankizumab proved beneficial in the treatment of moderate-to-severe psoriasis compared with a placebo and ustekinumab (**Supplementary Table**) (118).

Tildrakizumab is a humanized IgG1 monoclonal antagonist, targeting IL-23p19. The data from reSURFACE 1 and reSURFACE 2 have indicated significant safety and efficacy of tildrakizumab for the treatment of chronic plaque psoriasis; moreover, tildrakizumab was well-tolerated by the patients **(Supplementary Table**) (119). A pooled analysis of three trials showed that tildrakizumab maintains beneficial impact and low rates of serious TEAEs. The PASI75 scores of patients continuously treated with 100 mg and 200 mg tildrakizumab at 64 weeks were 86 and 83%, respectively (120).

Guselkumab, a human monoclonal anti-IL-23p19 antagonist, is used for the treatment of PsA (121). Two phase III trials (DISCOVER-1 and DISCOVER-2) showed that patients with PsA treated with guselkumab showed excellent and rapid, improvements in their condition; moreover, guselkumab treatment was safe for these patients. The results of the trials revealed that, at week 24, the ACR20 response rates were 52 and 64% for guselkumab administered every 4 weeks and 8 weeks, respectively (**Supplementary Table**) (122, 123).

In summary, the aforementioned clinical trials reported that anti-IL-23 antibodies can successfully control psoriasis and PsA. However, the long-term follow-up data on anti-IL-23p19 biologics are limited. Multicenter studies with large sample sizes should be conducted in the future to evaluate the long-term efficacy and safety of these antagonists.

## Hidradenitis Suppurativa

### Role of IL-17 in Hidradenitis Suppurativa

HS is a Th1/Th17-driven inflammatory skin disease (**Figure 2**) (124). The histopathological analysis of skin biopsy samples has revealed that the frequencies of the Th17 cells and regulatory T (Treg) cells are elevated in the lesional HS skin (19). The levels of inflammatory cytokines (IL-17, IL-23, IL-1β, TNF-α, and IL-12) are high in the lesional, perilesional, and uninvolved skin of patients with HS (125, 126). The serum levels of IL-17 and S100A8/A9 are higher in the patients with HS than in healthy individuals (127, 128).

The neutrophils and Th17 cells are the major sources of IL-17 in HS; in contrast, the keratinocytes are a key source of proinflammatory molecules, including S100A8/A9, NACHT, LRR, and PYD domains-containing protein 3, and caspase-1 (19, 129). Notably, HS showed histopathological changes characteristic of epidermal psoriasiform hyperplasia, follicular plugging, and infiltration of low-grade leucocytes in the uninvolved skin of perilesional HS (130). In such microenvironments, the IL-17-stimulated keratocytes show upregulation of the expression of the proinflammatory proteins (S100A8/A9) and promotion of the release of IL-1β by activation of the NACHT, LRR, and PYD domains-containing protein 3. Consequently, large amounts of proinflammatory molecules are recruited to promote the influx of the neutrophils, which, in turn, upregulate the release of IL-17 and S100A8/A9; thus, a positive-feedback loop of the inflammatory response is maintained (**Figure 2**) (129, 131).

### Targeted Therapy in Hidradenitis Suppurativa

An open-label and single-site exploratory trial has reported the efficacy of targeting IL-17A with secukinumab in the treatment of HS. The nine patients administrated of 300 mg secukinumab once a week from baseline for 5 weeks and then every 4 weeks. At 24 weeks, 67% patients with HS achieved Hidradenitis Suppurativa Clinical Response (HiSCR) score (132). Very recently, an open-label pilot cohort study on 10 patients assessed the well tolerability and clinical response of brodalumab in the treatment of moderate to severe HS. It demonstrated that patients received 210 mg brodalumab achieving HiSCR at week 12, and HiSCR improvement occurred as early as week 2 (133). An open-label study indicates that, at week 40, a moderate-to-marked improvement of the modified Sartorius score was achieved in 82% (14/17) of patients with HS receiving IL-12/23 biologic ustekinumab therapy (134). The data on the efficacy and safety of biologics for treating HS are limited, and further studies with adequate sample sizes are required to establish the effective and long-term impact of treatment.

## Atopic Dermatitis

Traditionally, AD was considered a Th2 immune response with elevated levels of IgE. Studies have revealed that the Th1, Th2, Th22, and Th17 cells are involved in the pathogenesis of AD (**Figure 2**) (135, 136). It has been demonstrated that Th22 and Th17 immune responses contribute to chronic skin lesions of AD, especially in pediatric, intrinsic, and Asian patients (137–140).

IL-17E (also called IL-25) level increases in the epidermis in patients with AD (141). In keratinocytes, the null mutation of filaggrin gene (FLG) is associated with the skin barrier dysfunction, increasing the risk of AD (142, 143). FLG synthesis is suppressed by IL-17E in the keratinocytes (144). Moreover, the data from mouse models indicated that IL-17E induces the type 2 innate lymphoid cells to produce type 2 cytokines (IL-5 and IL-13) (145).

To investigate the efficacy of anti-IL-17A biologics in AD, a randomized phase II trial was conducted involving 41 patients who were administered secukinumab. However, the trial results showed that at week 16, both clinical assessments (the Scoring Atopic Dermatitis index and Eczema Area and Severity Index) and lesional skin immunohistochemical analysis of patients receiving secukinumab revealed no significant improvement compared with those receiving a placebo (146). This trial demonstrated that IL17 is not a pivotal contributor to the pathogenesis of AD, even in the subsets of patients with higher Th17 activation.

Although ustekinumab showed promising efficacy in a review which included published case reports and case series (147), no efficacy was observed in randomized controlled trials of targeting IL-12/23 for treating patients with AD (148, 149). Additional studies with large sample sizes and may show the efficacy of ustekinumab in treating AD.

## Alopecia Areata

AA is a common inflammatory skin disorder, which is characterized by nonscarring hair loss *via* infiltration of the CD8^+^ T cells and increase in the levels of cytokines (IFN-γ, TNF-α, IL-17 and IL-4; **Figure 2**) (150). IL-2, IFN-γ, IL-10, IL-13, and IL-17A are expressed at high levels in the serum of patients with AA, while the level of transforming growth factor -β1 is decreased (151, 152). The Th17 cell frequencies and IL-17 levels significantly increased both in the peripheral blood and scalp lesions in patients with AA; however, the frequency of Treg cells decreased (153, 154). Studies have reported that patients with AA do not show any response to the administration of anti-IL-23/IL-12 ustekinumab (n = 4) or anti-IL-17A secukinumab (n = 7) (155, 156). Therefore, it cannot be concluded the contribution of Th17/IL-17 in the pathogenesis of AA. Further clinical trials with large sample size may reveal the value of IL-17 as a target of AA.

## Pityriasis Rubra Pilaris

PRP is a rare acquired inflammatory skin disease. The levels of Th17 and Th1 cytokines increase in the lesional skin of the patients with PRP, including IL-17A, IL-17F, IL-22, TNF, IL-6, IL-12, IL-23, and IL-1β (157). The IL-23/Th17 axis seems to be important in the pathogenesis of PRP due to the clinical and histopathologic improvement in the targeting IL-12/23 and IL-17A (ustekinumab, secukinumab, and ixekizumab) treatment of patients with PRP (157–160). In a single-armed trial, analyzing changes in the clinical signs and symptoms (using PASI scores) showed that PASI50, PASI75 and PASI90 response rates were 58, 42, and 17% respectively during ixekizumab treatment of PRP at week 24 (160). For those 5 patients who failed to conventional therapies, all of them have achieved clinical improvement from ustekinumab, particularly, changes in decreased erythema, follicular hyperkeratosis, and scaling during a 15-month follow-up period (161).

## Pemphigus

In the serum and lesional skin of patients with pemphigus vulgaris, the levels of IL-23 and IL-17 increase, both are significantly correlated with diseases severity (162, 163). The frequency of CD4^+^IL-17^+^cells and the level of IL-23R mRNA show increases in the serum of patients with pemphigus foliaceus (164), in contrast to showing decreases in newly diagnosed patients with pemphigus vulgaris (165). This may be a result of the Th17 cells have plasticity and converting to Th1-like Th17 cells (165–167). There are some reports that the frequency of Th17 cells and level of IL-17 show decreases in other autoimmune and inflammatory diseases, such as lipopolysaccharides responsive beige-like anchor protein deficiency (165, 167).

## Systemic Sclerosis

The imbalance and dysfunction of Th17/Treg cells are crucial to the generation of SSc (168). Quantitative analysis of Th17 cytokines in lesional skin of SSc showed that the expression of IL-17A, IL-13, IL-22, and IL-26 mRNA are higher compared with healthy control (169, 170). The levels of circulating Th17 cells and IL-17 elevated in serum of patients with SSc. They are in correlation with disease severity and collagen overproduction (171, 172). The elevated levels of IL-17A act on dermal vascular smooth muscle cells to promote vascular fibrosis in the patients with SSc, *via* activating extracellular signal-regulated kinase 1/2 signaling pathway (173).

## Conclusion

In summary, the Th17/IL-17 axis has been identified as a key factor in skin inflammatory diseases, such as psoriasis, HS, AD, PRP, pemphigus, and SSc. Neutralizing IL-17 or IL-23 in psoriasis, HS and PRP has shown promising clinical improvements. Additional studies are required to identify whether IL-17 is involved in the pathogenesis of AA, PRP, pemphigus, and SSc, which may lead to the development of targeted strategies for efficiently ameliorating or specifically eliminating these debilitating diseases.

## Author Contributions

TL, YL, JQ, and HF contributed to conception, literature search, and manuscript writing. SL, SY, ST, and YD created graphical illustrations. TL, JQ, and HF contributed to manuscript revision and read the submitted version. All authors contributed to the article and approved the submitted version.

## Funding

This work was supported by the National Natural Science Foundation of China (81972931 to HF), the Medical and Health Science and Technology Project of Health Commission of Zhejiang Province (2020KY558 to JQ), and the Zhejiang Provincial Natural Science Foundation (LY20H110001 to YL).

## Conflict of Interest

The authors declare that the research was conducted in the absence of any commercial or financial relationships that could be construed as a potential conflict of interest.
