# Supplementary material for: The IL-23/IL-17 Pathway in Inflammatory Skin Diseases: From Bench to Bedside
Source: Front Immunol. 2020 Nov 17;11:594735. doi: 10.3389/fimmu.2020.594735 (PMC7705238; doi:10.3389/fimmu.2020.594735)
Supplement: Supplementary file 1 [file DataSheet_1.pdf]

Supplementary Table| Clinical trials of targeting IL-17 pathway for treating psoriasis

Target	Name	Disease	Treatment	Response rate observed in trial, %					
				PASI75	PASI90	PASI100	ACR20	ACR50	ACR70
IL-17A	Secukinumab (FUTURE 2)<br><br>III(1)	psoriatic arthritis	Secukinumab 300 mg 24W	63	49	-	54	35	-
			Secukinumab 150 mg 24W	48	33	-	51	35	-
			Secukinumab 75 mg 24W	28	12	-	29	18	-
			Placebo 24W	16	9	-	15	7	-
	Secukinumab (ERASURE)<br><br>III(2)	moderate-to-severe plaque psoriasis	Secukinumab 300 mg 12W	81.6	59.2	28.6			
			Placebo 12W	4.5	1.2	0.8			
			Secukinumab 150 mg 12W	71.6	39.1	12.8			
			Secukinumab 300 mg 52W	80.5	-	-			
			Secukinumab 150 mg 52W	72.4	-	-			
	Secukinumab (FIXTURE)<br><br>III(2)	moderate-to-severe plaque psoriasis	Secukinumab 300 mg 12W	77.1	54.2	24.1			
			Secukinumab 150 mg 12W	67	41.9	14.4			
			placebo	4.9	1.5	-			

	Ixekizumab (SPIRIT-P1)<br><br>III(3)	psoriatic arthritis	Ixekizumab 80 mg 12W (Q2W)	69.5	57.6	40.7	60.2	39.8	16.5
			Ixekizumab 80 mg 12W (Q4W)	75.3	52.1	31.5	57	33.6	15
			Placebo 12W	7.5	1.5	1.5	31.1	4.7	0
			Ixekizumab 80 mg 24W (Q2W)	79.7	67.8	52.5	62.1	46.6	34
			Ixekizumab 80 mg 24W (Q4W)	71.2	56.2	42.5	57.9	40.2	23.4
			Placebo 24W	10.4	6.0	3.0	30.2	15.1	5.7
	Ixekizumab (SPIRIT-P2)<br><br>III(4)	psoriatic arthritis	Ixekizumab 80 mg 24W (Q2W)	60	50	28	48	33	12
			Ixekizumab 80 mg 24W (Q4W)	56	44	35	53	35	22
			Placebo	15	12	4	19	5	0
	Ixekizumab (UNCOVER-3)<br><br>III(5)	moderate-to-severe plaque psoriasis	Ixekizumab (156W)	97.2	86.7	63.9			
IL - 17RA	Brodalumab (AMAGINE-1)<br><br>III (6, 7)	moderate-to-severe plaque psoriasis	Brodalumab 210mg 12W	83.3	70.3	41.9			
			Placebo 12W	2.7	0.9	0.5			
			Brodalumab 210mg 52W	98.7	86.8	73.7			

			Brodalumab 210mg 120W	95.7	84.1	73.9			
	Brodalumab<br><br>(8)	GPP, PsE	Brodalumab 140mg 12W (GPP)	58.3	33.3	25			
			Brodalumab 140mg 12W (PsE)	77.8	50.0	16.7			
IL－17A/F	Bimekizumab (BE ABLE 1)<br><br>II(9)	moderate-to-severe plaque psoriasis	Bimekizumab 64 mg 12W	61.5	46.2	28.2			
			Bimekizumab 160 mg 12W	81.4	67.4	27.9			
			Bimekizumab 320 mg 12W	93	79.1	55.8			
			Bimekizumab 480 mg 12W	83.7	72.1	48.8			
			placebo	4.8	0	0			
	Bimekizumab (BE ACTIVE)<br><br>II(10)	active psoriatic arthritis	Bimekizumab 160 mg 12W	64	46	36	73	41	20
			Bimekizumab 320 mg 12W	73	54	38	51	24	15
			Placebo 12W	7	7	7	19	7	5
			Bimekizumab 160 mg 24W	81	67	63	68	60	33
			Bimekizumab 320 mg 24W	81	77	58	80	54	27
			Bimekizumab 160 mg 48W	78	70	67	70	55	43
			Bimekizumab 320 mg 48W	85	85	73	76	63	39

IL-12/IL-23p40	Ustekinumab (PHOENIX 1)<br><br>III (11)	moderate-to-severe plaque psoriasis	Ustekinumab 45 mg 12W	67.1	41.6	12.5			
			Ustekinumab 90 mg 12W	66.4	36.7	10.9			
			Placebo 12W	3.1	2.0	0.0			
			Ustekinumab 45 mg 28W	71.2	49.2	20.8			
			Ustekinumab 90 mg 28W	78.6	55.6	29.2			
	Ustekinumab (PHOENIX 2)<br><br>III (12)	moderate-to-severe plaque psoriasis	Ustekinumab 45 mg 12W	66.7	42.3	18.1			
			Ustekinumab 90 mg 12W	75.7	50.9	18.2			
			Placebo 12W	3.7	0.7	0			
			Ustekinumab 45 mg 28W	69.5	44.8	18.6			
			Ustekinumab 90 mg 28W	78.5	54.3	29.5			
IL－23p19	Risankizumab (UltIMMa-1)<br><br>III(13)	moderate-to-severe plaque psoriasis	Risankizumab 150 mg 16W	-	75.3	35.9			
			Placebo 16W	-	4.9	0			
			Risankizumab 150 mg 52W	-	81.9	56.3			
	Risankizumab<br><br>(UltIMMa-2)<br><br>III(13)	moderate-to-severe plaque psoriasis	Risankizumab 150 mg 16W	-	74.8	50.7			
			Placebo 16W	-	2.0	2.0			

			Risankizumab 150 mg 52W	-	80.6	59.5			
	Tildrakizumab (reSURFACE 1)<br><br>III(14)	moderate-to-severe plaque psoriasis	Tildrakizumab 100 mg 12W	64	35	14			
			Tildrakizumab 200 mg 12W	62	35	14			
			Placebo 12W	6	3	1			
			Tildrakizumab 100 mg 28W	80	52	24			
			Tildrakizumab 200 mg 28W	82	59	32			
	Tildrakizumab (reSURFACE 2)<br><br>III(14)	moderate-to-severe plaque psoriasis	Tildrakizumab 100 mg 12W	61	39	12			
			Tildrakizumab 200 mg 12W	66	37	12			
			Placebo 12W	6	1	0			
			Tildrakizumab 100 mg 28W	-	56	23			
			Tildrakizumab 200 mg 28W	-	58	27			
	Guselkumab (DISCOVER-1)<br><br>III(15)	psoriatic arthritis	Guselkumab 100 mg 24W (Q4W)	86	63	45	59	36	20
			Guselkumab 100 mg 24W (Q8W)	76	50	26	52	30	12

			Placebo 24W	14	12	6	22	9	6
	Guselkumab (DISCOVER-2)<br><br>III(16)	psoriatic arthritis	Guselkumab 100 mg 24W (Q4W)	78	61	45	64	33	13
			Guselkumab 100 mg 24W (Q8W)	79	69	45	64	31	19
			Placebo 24W	23	10	3	33	14	4

References

1. McInnes IB, Mease PJ, Kirkham B, Kavanaugh A, Ritchlin CT, Rahman P, et al. Secukinumab, a human anti–interleukin–17A monoclonal antibody, in patients with psoriatic arthritis (FUTURE 2): a randomised, double–blind, placebo–controlled, phase 3 trial. *The Lancet* (2015) 386(9999):1137–46. doi: 10.1016/s0140–6736(15)61134–5.

2. Langley RG, Elewski BE, Lebwohl M, Reich K, Griffiths CE, Papp K, et al. Secukinumab in plaque psoriasis––results of two phase 3 trials. *N Engl J Med* (2014) 371(4):326–38. Epub 2014/07/10. doi: 10.1056/NEJMoa1314258. PubMed PMID: 25007392.

3. Mease PJ, van der Heijde D, Ritchlin CT, Okada M, Cuchacovich RS, Shuler CL, et al. Ixekizumab, an interleukin–17A specific monoclonal antibody, for the treatment of biologic–naive patients with active psoriatic arthritis: results from the 24–week randomised, double–blind, placebo–controlled and active (adalimumab)–controlled period of the phase III trial SPIRIT–P1. *Ann Rheum Dis* (2017) 76(1):79–87. Epub 2016/08/25. doi: 10.1136/annrheumdis–2016–209709. PubMed PMID: 27553214; PubMed Central PMCID: PMCPMC5264219.

4. Nash P, Kirkham B, Okada M, Rahman P, Combe B, Burmester GR, et al. Ixekizumab for the treatment of patients with active psoriatic arthritis and an inadequate response to tumour necrosis factor inhibitors: results from the 24–week randomised, double–blind, placebo–controlled period of the SPIRIT–P2 phase 3 trial. *Lancet* (2017) 389(10086):2317–27. Epub 2017/05/30. doi: 10.1016/s0140–6736(17)31429–0. PubMed PMID: 28551073.

5. Leonardi C, Maari C, Philipp S, Goldblum O, Zhang L, Burkhardt N, et al. Maintenance of skin clearance with ixekizumab treatment of psoriasis: Three–year results from the UNCOVER–3 study. *J Am Acad Dermatol* (2018) 79(5):824–30.e2. Epub 2018/05/29. doi: 10.1016/j.jaad.2018.05.032. PubMed PMID: 29803904.

6. Papp K, Menter A, Leonardi C, Soung J, Weiss S, Pillai R, et al. Long–term efficacy and safety of brodalumab in psoriasis through 120 weeks and after withdrawal and retreatment: subgroup analysis of a randomized phase III trial (AMAGINE–1). *Br J Dermatol* (2020). Epub 2020/04/15. doi: 10.1111/bjd.19132. PubMed PMID: 32286683.

7. Papp KA, Reich K, Paul C, Blauvelt A, Baran W, Bolduc C, et al. A prospective phase III, randomized, double–blind, placebo–controlled study of brodalumab in patients with moderate–to–severe plaque psoriasis. *Br J Dermatol* (2016) 175(2):273–86. Epub 2016/02/26. doi: 10.1111/bjd.14493. PubMed PMID: 26914406.

8. Yamasaki K, Nakagawa H, Kubo Y, Ootaki K. Efficacy and safety of brodalumab in patients with generalized pustular psoriasis and psoriatic erythroderma: results from a 52–week, open–label study. *Br J Dermatol* (2017) 176(3):741–51. Epub 2016/10/22. doi: 10.1111/bjd.14702. PubMed PMID: 27106510.

9. Papp KA, Merola JF, Gottlieb AB, Griffiths CEM, Cross N, Peterson L, et al. Dual neutralization of both interleukin 17A and interleukin 17F with bimekizumab in patients with psoriasis: Results from BE ABLE 1, a 12–week randomized, double–blinded, placebo–controlled phase 2b trial. *J Am Acad Dermatol* (2018) 79(2):277–86.e10. Epub 2018/04/03. doi: 10.1016/j.jaad.2018.03.037. PubMed PMID: 29609013.

10. Ritchlin CT, Kavanaugh A, Merola JF, Schett G, Scher JU, Warren RB, et al. Bimekizumab in patients with active psoriatic arthritis: results from a 48–week, randomised, double–blind, placebo–controlled, dose–ranging phase 2b trial. *The Lancet* (2020) 395(10222):427–40. doi: 10.1016/s0140–6736(19)33161–7.

11. Leonardi CL, Kimball AB, Papp KA, Yeilding N, Guzzo C, Wang Y, et al. Efficacy and safety of ustekinumab, a human interleukin–12/23 monoclonal antibody, in patients with psoriasis: 76–week results from a randomised, double–blind, placebo–controlled trial (PHOENIX 1). *Lancet* (2008) 371(9625):1665–74. Epub 2008/05/20. doi: 10.1016/s0140–6736(08)60725–4. PubMed PMID: 18486739.

12. Papp KA, Langley RG, Lebwohl M, Krueger GG, Szapary P, Yeilding N, et al. Efficacy and safety of ustekinumab, a human interleukin–12/23 monoclonal antibody, in patients with psoriasis: 52–week results from a randomised, double–blind, placebo–controlled trial (PHOENIX 2). *Lancet* (2008) 371(9625):1675–84. Epub 2008/05/20. doi: 10.1016/s0140–6736(08)60726–6. PubMed PMID: 18486740.

13. Gordon KB, Strober B, Lebwohl M, Augustin M, Blauvelt A, Poulin Y, et al. Efficacy and safety of risankizumab in moderate–to–severe plaque psoriasis (UltIMMa–1 and UltIMMa–2): results from two double–blind,

randomised, placebo-controlled and ustekinumab-controlled phase 3 trials. *Lancet* (2018) 392(10148):650-61. Epub 2018/08/12. doi: 10.1016/s0140-6736(18)31713-6. PubMed PMID: 30097359.
14. Reich K, Papp KA, Blauvelt A, Tyring SK, Sinclair R, Thaçi D, et al. Tildrakizumab versus placebo or etanercept for chronic plaque psoriasis (reSURFACE 1 and reSURFACE 2): results from two randomised controlled, phase 3 trials. *Lancet* (2017) 390(10091):276-88. Epub 2017/06/10. doi: 10.1016/s0140-6736(17)31279-5. PubMed PMID: 28596043.
15. Deodhar A, Helliwell PS, Boehncke WH, Kollmeier AP, Hsia EC, Subramanian RA, et al. Guselkumab in patients with active psoriatic arthritis who were biologic-naive or had previously received TNFα inhibitor treatment (DISCOVER-1): a double-blind, randomised, placebo-controlled phase 3 trial. *Lancet* (2020) 395(10230):1115-25. Epub 2020/03/18. doi: 10.1016/s0140-6736(20)30265-8. PubMed PMID: 32178765.
16. Mease PJ, Rahman P, Gottlieb AB, Kollmeier AP, Hsia EC, Xu XL, et al. Guselkumab in biologic-naive patients with active psoriatic arthritis (DISCOVER-2): a double-blind, randomised, placebo-controlled phase 3 trial. *Lancet* (2020) 395(10230):1126-36. Epub 2020/03/18. doi: 10.1016/s0140-6736(20)30263-4. PubMed PMID: 32178766.
